# TRPM2 Promotes Lipophagy Through TFEB and LAL in HFD-Fed Mice

**DOI:** 10.3390/cells15151361

**Published:** 2026-07-28

**Authors:** Fan Ying, Duan Zhuo, Shaobo Zhou, Liwen Jiang, Xiaoqiang Yao

**Affiliations:** 1 School of Biomedical Sciences, Faculty of Medicine, The Chinese University of Hong Kong, Hong Kong SAR, China; 2 Heart and Vascular Institute and Li Ka Shing Institute of Health Science, Faculty of Medicine, The Chinese University of Hong Kong, Hong Kong SAR, China; 3 Southern University of Science and Technology Yantian Hospital, Shenzhen 518081, China; ssophiaz@163.com (D.Z.); zhoushaobodoctor@sina.com (S.Z.); 4 Centre for Cell and Developmental Biology, State Key Laboratory of Agrobiotechnology, School of Life Sciences, The Chinese University of Hong Kong, Hong Kong SAR, China; ljiang@cuhk.edu.hk

**Keywords:** TRPM2 channels, lipophagy, lysosomal biogenesis, lysosomal acid lipase

## Abstract

**Highlights:**

This study identified a novel protective role of TRPM2-mediated lipophagy in hepatic steatosis under high-fat diet. This action is mediated through TFEB and lysosomal acid lipase.

**Abstract:**

An abnormality of Ca^2+^ signaling may aggravate lipid accumulation in steatotic hepatocytes, leading to non-alcoholic fatty liver disease. However, the molecular identity of Ca^2+^-permeable channels and the mechanism of involvement of these channels in steatotic hepatocytes are not well-studied. In the present study, we investigated the role of a Ca^2+^-permeable channel TRPM2 in lipid metabolism in steatotic hepatocytes. A mouse model of non-alcoholic fatty liver disease was established by high-fat-diet feeding. Fat accumulation, fibrosis, lipophagic indexes, TFEB and lysosomal acid lipase in the liver tissue and/or hepatocytes were compared between *TRPM2*-knockout mice and wild-type mice. Knockout of the *TRPM2* gene aggravated liver fat accumulation and fibrosis. Mechanistically, the *TRPM2* knockout impaired the lipophagic process, decreased lysosomal abundance and attenuated lysosomal/autolysosomal acidification in mouse hepatocytes. Furthermore, the *TRPM2* knockout reduced TFEB expression and its nuclear translation and also reduced the expression/activity of lysosomal acid lipase. These data demonstrate that *TRPM2* deficiency may reduce lipophagy via its action on TFEB and lysosomal acid lipase, consequently contributing to liver steatosis and NAFLD under high-fat feeding conditions.

## 1. Introduction

Non-alcoholic fatty liver disease (NAFLD), also called metabolic dysfunction-associated steatotic liver disease (MASLD), is the most common cause of chronic liver disease, affecting 20–40% of adults worldwide. It is a spectrum of liver pathology characterized by hepatic steatosis, where the main feature is excessive fat deposition within hepatocytes, liver inflammation and fibrosis [1,2]. Despite its high prevalence, the detailed regulatory mechanism of MASLD/NAFLD is still unclear.

Autophagy is a critical cellular degradation pathway that delivers cytoplasmic cargo into lysosomes for degradation, contributing to recycling of long-lived proteins and clearance of damaged organelles [3]. Lipophagy is a type of selective autophagy in which lysosomes degrade lipid droplets. In the lipophagic process, lipid droplets are selectively sequestered in the autophagosomes and delivered into the lysosomes for degradation by lysosomal acid lipases [4,5]. In MASLD/NAFLD, lipophagy is blocked due to reduced autophagosome/lysosome fusion, decreased numbers of functional lysosomes and decreased lysosomal acidity [6]. Dysfunctional lipophagy further enhances intracellular lipid droplet accumulation, eventually leading to the development of hepatic steatosis [6]. Therefore, hepatic lipophagy plays a protective role in MASLD/NAFLD.

Previous studies have shown that endolysosomal cation channel TRPML1 may mediate endolysosomal Ca^2+^ release, subsequently promoting autophagy via facilitating multiple steps of autophagy, including autophagy induction [7], autophagosome–lysosome fusion [8], and autolysosome degradation [9]. Mechanistically, TRPML1-mediated endolysosomal Ca^2+^ release enhances the nuclear translocation of transcription factor TFEB, consequently activating autophagy [10]. A recent study also suggests that TRPML1 can regulate lipophagy by mediating fatty acid efflux in hepatocytes [11].

TRPM2 is a Ca^2+^-permeable cation channel that is widely expressed in a variety of organs/tissues/cell types [12,13,14]. At the subcellular level, TRPM2 is not only localized to the plasma membrane but also expressed in the lysosomal membrane, where it functions as a lysosomal Ca^2+^ release channel [15]. Previous studies from us and others have reported that TRPM2 may regulate autophagy in several cell types, including pericytes, gastric cancer cells, vascular smooth muscle cells and hepatocytes [15,16,17,18,19]. However, up to now, there has been no report investigating the role of TRPM2 in lipophagy. In the present study, we studied the role of TRPM2 in lipophagy during lipid overload in hepatocytes. The results showed that *TRPM2*-knockout mice exhibited exaggerated hepatic steatosis and fibrosis after a high-fat diet (HFD). The underlying mechanism involves a decrease in hepatocyte lipophagy after *TRPM2* knockout, causing accumulation of lipid droplets in hepatocytes. These findings uncovered a novel mechanism that *TRPM2* deficiency may impair lipophagy in hepatocytes, consequently contributing to liver steatosis and MASLD/NAFLD under high-fat feeding conditions.

## 2. Materials and Methods

### 2.1. Animal Experiments

*TRPM2*^−/−^ mice and *TRPM2*^+/+^ littermates were kindly provided from Yasue Mori Group in Kyoto University in Japan and were maintained in a C57BL/6J background [20]. The *TRPM2* gene was disrupted in the *TRPM2*^−/−^ mice by deleting the exon that contributes to the putative pore region of the TRPM2. All mice were housed in the Laboratory Animal Services Centre of the Chinese University of Hong Kong on a 12 h light–dark cycle, with fresh water and food. To develop the non-alcoholic fatty liver model, six-week-old male mice were either fed a normal diet (ND) (24.7% protein, 13.2% fat, 62.1% carbohydrate; LabDiet PicoLab Rodent Diet, Purina Nutrition International; Brentwood, MO, USA) or a high-fat diet (HFD) (20% protein, 60% fat, 20% carbohydrate; Research Diets, D12492; New Brunswick, NJ, USA). The allocation of the mice was random. All animal experiments were approved by the Hong Kong Department of Health and the Animal Experimentation Ethics Committee of the Chinese University of Hong Kong (Ref No. 18-048-HMF, approved 28 March 2018).

### 2.2. Dissection

The mice were terminated by CO_2_ asphyxiation. The blood was taken from the celiac vein within 1 min. Liver tissues were collected and used immediately or snap-frozen in liquid nitrogen for further analysis. This included autophagic and lipophagic analysis and tissue sectioning followed by histochemical staining.

### 2.3. Measurements of Plasma Components

Plasma was collected by centrifuging the whole blood within heparin-coated tubes (LEOPharma, Ballerup, Denmark) at 3000 g at room temperature for 10 min. Commercial kits for ALT, AST and cholesterol detection were used to determine ALT, AST, and total cholesterol quantitatively (Stanbio, Boerne, TX, USA).

### 2.4. Histological Staining

Fresh liver tissues were fixed in 4% formaldehyde for 48 h and immersed at 70% ethanol overnight before paraffin embedment. Serial 5 µm sections were obtained. To assess hepatic injury, liver sections were stained with H&E, Masson trichrome and Sirius Red. To stain lipids on liver sections, tissues were fixed in optimal cutting temperature compounds (VWR International, Visalia, CA, USA). Eight µm-thick sections were cut and mounted onto the slides. After air drying, the slides were firstly fixed in 4% formaldehyde and washed with PBS. The slides were then placed in 60% isopropanol for 10 min and stained in 0.3% Oil Red O solution for 15–30 min, then transferred into 60% isopropanol for 5 min. After rinsing with distilled H_2_O, the slides were processed for hematoxyline counterstaining. Photoimages were taken under a light macroscope.

### 2.5. Primary Hepatocyte Isolation and Culture

The mice were anesthetized with 80 mg/kg + 5 mg/xylazine (i.p.) for 5 min; the portal vein was cannulated and then perfused for 5 min with a Perfusion Buffer (PBS containing 10 mM Glucose, 0.5 mM EGTA, 25 mM HEPES, pH 7.5), followed by a Digest Buffer (Perfusion Buffer plus 10 mM CaCl_2_, 0.05% Type IV collagenase) pre-warmed to 37 °C. The liver was removed after the digestion, followed by mechanical disassociation of hepatocytes and filtration through a 70 µm filter. The cells were purified with a density gradient centrifuge and then cultured with a culture medium (DMEM HG, 100 nM insulin, 100 nM dexamethasone).

### 2.6. Cytosolic Ca^2+^ Measurement

Freshly isolated primary hepatocytes were seeded in a confocal dish. After 24–48 h, the cells were loaded with Fluo4/AM (1 μM, Invitrogen, Carlsbad, CA, USA) in the dark for 30 min at 37 °C, then washed twice with PBS. The cells were buffered in Tyrode’s solution containing, in mM, NaCl 140, KCl 5.4, CaCl_2_ 1.8, MgCl_2_ 1, glucose 10, and HEPES 10, adjusted to pH 7.40 with NaOH. In some experiments, 200 μM Gly-Phe β-naphthylamide (GPN) (Selleck, Houston, TX, USA) was also included to interrupt lysosome membrane integrity. A total of 3 mM H_2_O_2_ was added to activate TRPM2. Data were acquired using a confocal microscope (Olympus FV1000, Olympus, Tokyo, Japan). At least 30 cells were recorded for each experiment. Changes in cytosolic Ca^2+^ were calculated as a ratio of fluorescence relative to the average intensity before stimulation (F1/F0).

### 2.7. Real-Time PCR

RNA was extracted using a QIAzol lysis reagent (Qiagen, Santa Clarita, CA, USA) and further purified using the RNeasy mini kit (Qiagen) following the manufacturer’s instructions. Real-time quantitative PCRs were performed as described by Ying et al. [21]. The sequences of the oligonucleotide primers used for PCR amplification were LCAD-forward 5′-GGAAGCTGCATAAGATGGGA-3′; LCAD-reverse 5′-GAAATCGCCAACTCAGCAAT-3′; MCAD-forward 5′-GCTCGTGAGCACATTGAAAA-3′; MCAD-reverse 5′-CATTGTCCAAAAGCCAAACC-3′; CD36-forward 5′-TCGGAACTGTGGGCTCATTG-3′; CD36-reverse 5′-CCTCGGGGTCCTGAGTTATATTTTC-3′; TFEB-forward 5′-AACAAAGGCACCATCCTCAA-3′; TFEB-reverse 5′-GGAGCCAGAGCTGCTTGTTA-3′; ATG3-forward 5′-TCA CAACACAGGTATTACAG-3′; ATG3-reverse 5′-CTTCCTCGTCTTCTTCATC-3′; ATG5-forward 5′-ACTTGCTTTACTCTCTATCAG-3′; ATG5-reverse 5′-CATCTTCTTGTCTCATAACCT-3′; ATG7-forward 5′-CAGAAGAAGTTGAACGAGTA-3′; ATG7-reverse 5′-CAGAGTCACCATTGTAGTAAT-3′; LIPA-forward 5′-GAACACTCGGTCCTGACAGGAGAT-3′; LIPA-reverse 5′-AAGCCGTGCTGAAGATACACAACT-3′; β-actin-forward 5′-CCTGAGCGCAAGTACTCTGTGT-3′; and β-actin-reverse 5′-GCTGATCCACATCTGCTGGAA-3′. The mRNA levels of the various genes were normalized to *β-actin*, which was used as a reference gene in all experiments.

### 2.8. Protein Extraction and Western Blotting

Liver tissue or cells were homogenized in a RIPA lysis buffer with a cocktail of protease inhibitors [21]. The protein concentrations were determined by a Bio-Rad ABS kit. A total of 20 μg of the protein samples was loaded into each lane and separated by a 12% SDS-PAGE gel, then blotted to a PVDF membrane for detection with appropriate antibodies. Primary antibodies were against LC3I/II (1:1000, NB100-2220, Novus, St. Charles, MO, USA), P62/SQSTM1 (1:1000, ab91526, Abcam, Cambridge, UK), LAMP1 (1:1000, #MA1-164, Invitrogen), LAMP2 (1:1000, #PA1-655, Invitrogen), Cathepsin D (1:1000, 21327-1-AP, Proteintech, Rosemont, IL, USA), Raptor (1:1000, #2280, Cell Signaling, Danvers, MA, USA), TFEB (1:1000, 13372-1-AP, Proteintech), LIPA (1:1000, 12956-1-AP, Proteintech), GAPDH (1:3000, Cell Signaling), and PCNA (1:3000, 10205-2-AP, Proteintech). Horseradish peroxidase-conjugated secondary antibodies (1:3000, Cell Signaling) were followed by an ECL detection system (Amersham Pharmacia, Uppsala, Sweden).

### 2.9. PA and BSA Treatment of the Primary Cultured Hepatocytes

PA was complexed with BSA, as described elsewhere [22]. Briefly, palmitic acid powder was added to a 10% solution of fatty acid-free BSA and dissolved by shaking gently overnight at 37 °C to yield an 8 mM stock solution of palmitic acid complexed to BSA. The final molar ratio of free fatty acid to BSA was 5.2:1. Both PA and BSA (as a control) were added to culture medium DMED, and the primary cultured hepatocytes were exposed to 250 µM palmitic acid or 250 µM BSA as a control for 24 h.

### 2.10. Electron Microscopy

Electron microscopy was performed as described elsewhere [21]. Briefly, blocks of liver tissue were fixed with 2.5% glutaraldehyde and postfixed with 1% osmium tetroxide in a cacodylate buffer (100 mM sodiumcacodylate–HCl, pH 7.4). The tissues were then dehydrated and infiltrated, followed by embedding in epoxy resin with polymerization. Sections were stained with uranyl acetate and lead citrate and photographed with a transmission electron microscope (CM100; Philips Electron Optics, Eindhoven, The Netherlands). The images were obtained with 3000× magnification and 10,000× magnification.

### 2.11. Separation of Cellular Fractions

The cytoplasmic and nuclear fractions of the liver tissues were separated by using a commercial nuclear extraction kit (Millipore, Billerica, MA, USA).

### 2.12. Immunohistochemical and Immunofluorescence Staining

In some experiments, slides sectioned from paraffin blocks were deparaffinized in xylene and rehydrated in graded alcohols. A Tris-EDTA buffer was used for the antigen retrieval process using a standard microwave heating technique. After rinsing with 3% hydrogen peroxide, the slides were immersed in a BSA buffer. The antibodies used for immunostaining included TFEB (1:100, 13372-1-AP, Proteintech) and LIPA (1:100, 12956-1-AP, Proteintech). Positive signals were visualized by using a DAB solution. After staining of the nuclei with hematoxyline, the slides were examined under light macroscope.

In some other experiments, cryosections were prepared from frozen liver tissue or cells, fixed with 4% formaldehyde, and then incubated with 3% H_2_O_2_ for another 10 min. Sections were washed with tris-buffered saline (TBS) plus 0.025% Triton X-100 and blocked in 5% BSA in TBS for 30 min at room temperature. Diluted primary antibodies were used to incubate the tissue sections or cells for 30 min at 37 °C or overnight at 4 °C. Sections or cells were washed in TBS, followed by incubation with fluorescence-conjugated secondary antibodies in the dark for 1 h at 37 °C. After washing three times, DAPI was finally used for another 10 min. Images in five random visual fields were acquired with an Olympus FV1000 confocal microscope system.

### 2.13. Flow-Cytometric Measurement of Lysosomal Enzyme Activity

Primary hepatocytes were collected and stained with LysoLive LipaGreen (abcam, ab253380) according to the manufacturer’s instructions. Briefly, cells were washed twice with PBS and then stained using LysoLive LipaGreen (1:1000 in serum free medium) at 37 °C with 5% CO_2_ for 3 h. The cells were then harvested using 1% trypsin and resuspended with a FACS buffer (1% BSA and 20 mM HEPES in PBS, pH 7.4). Finally, the cells were run with a Flow cytometer using a 488 nm laser and FL-1 detection (~520 nm emission). The gates were set to exclude cellular debris and dead cells. At least 10,000 live cell events were captured. The median FL-1 signal was measured on a gated population of viable cells.

### 2.14. Lysosomal Acidification Measurement

LysoSensor™ Green DND-189 (Invitrogen) was used to measure the lysosomal acidification in the primary cultured hepatocytes. The primary cells were incubated with 1 μM of the LysoSensor probe for 30 min at 37 °C in the dark. Fluorescence was detected by an Olympus FV1000 confocal microscope system. Green puncta with strong fluorescence intensity represented lysosomes with low pH values.

### 2.15. Statistical Analysis

Data are expressed as means ± SD or means ± SEM. All statistical analysis was performed using GraphPad Prism software 5.0 (San Diego, CA, USA). The two-tailed unpaired Student’s *t* test, one-way ANOVA followed by Tukey’s test or two-way ANOVA followed by Bonferroni’s post-hoc test, wherever appropriate, were used for comparisons between experimental groups. Differences were statistically significant when *p* was less than 0.05.

## 3. Results

### 3.1. TRPM2 Gene Knockout Aggravated HFD-Induced Hepatic Steatosis and Fibrosis

**Figure 1:**
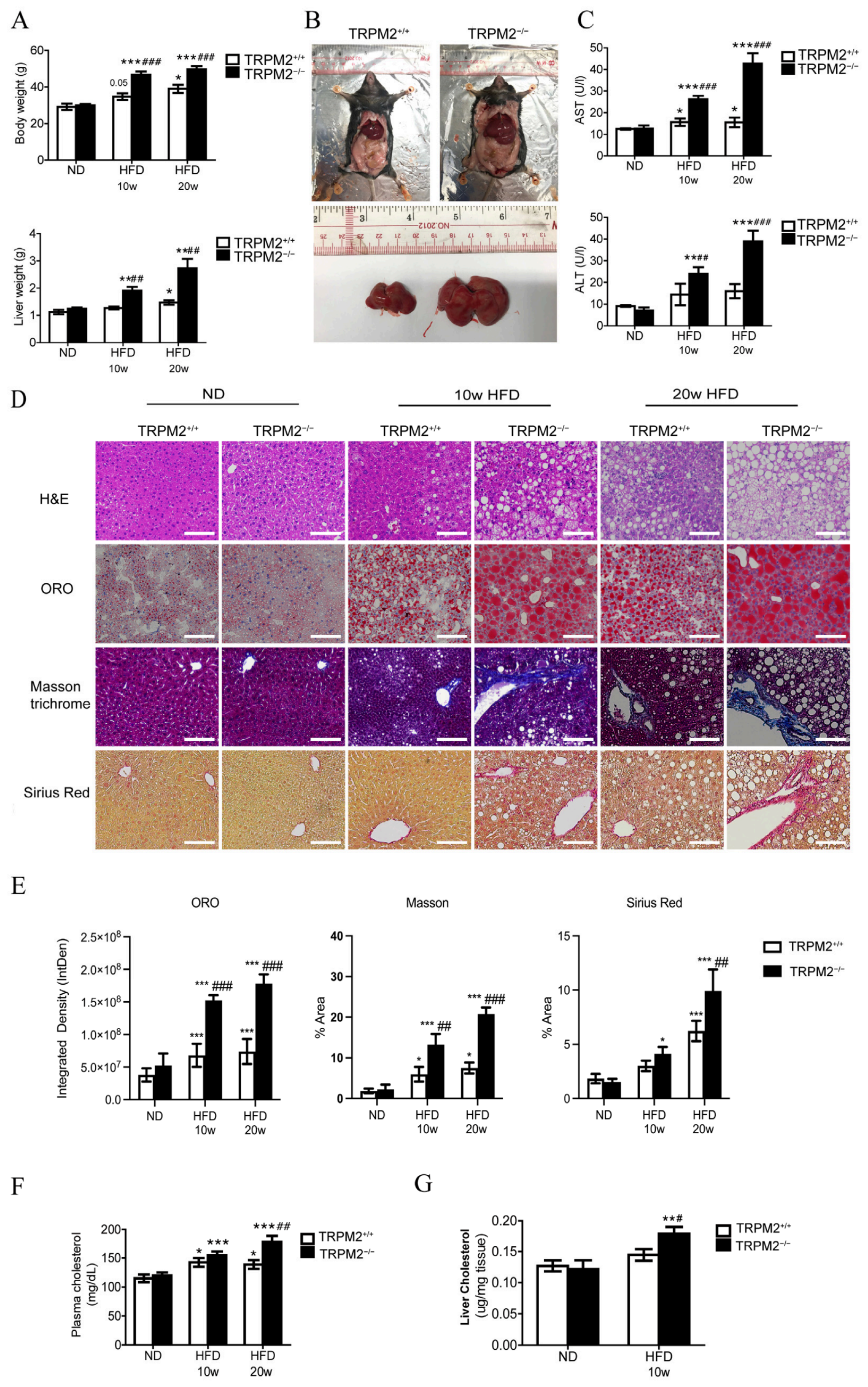
*TRPM2* gene knockout aggravated HFD-induced hepatic steatosis and fibrosis. (**A**) Comparison of body weight and liver weight between *TRPM2*^+/+^ and *TRPM2*^−/−^ mice fed with an ND or HFD for 10 weeks or 20 weeks. (**B**) Representative photographs of liver tissues in HFD-fed *TRPM2*^+/+^ and *TRPM2*^−/−^ mice for 10 weeks. (**C**) Plasma AST and ALT levels in ND-fed and HFD-fed *TRPM2*^+/+^ and *TRPM2*^−/−^ mice. (**D**) Representative photographs of thin liver sections of *TRPM2*^+/+^ and *TRPM2*^−/−^ mice fed with an ND or HFD for 10 weeks or 20 weeks. H&E staining illustrates the entirety of the morphological changes in the liver tissue; Oil Red O staining shows lipid droplet development; and Masson’s trichrome and Sirius red staining show collagen deposit in the fibrotic scar. (**E**) Statistic analyses of Oil Red O, Masson and Sirius red staining results. (**F**,**G**) Plasma cholesterol levels (**F**) and liver tissue cholesterol levels (**G**) of *TRPM2*^+/+^ and *TRPM2*^−/−^ mice fed with an ND or HFD for 10 weeks or 20 weeks. Data are shown as means ± SD in A and E and means ± SEM in C, F and G. Scale bar: 100 µm. * *p* < 0.05, ** *p* < 0.01, *** *p* < 0.001 ND vs. HFD; # *p* < 0.05, ## *p* < 0.01, ### *p* < 0.001 *TRPM2*^+/+^ vs. *TRPM2*^−/−^. *N* = 7–10 mice in (**A**–**C**,**F**,**G**); representative of *N* = 5 mice, 10 slides per mouse in (**D**,**E**).

A mouse model of MASLD/NAFLD was established by an HFD [23]. We compared the body weight and liver weight between wild-type (*TRPM2*^+/+^) and *TRPM2*-knockout (*TRPM2*^−/−^) male mice after normal diet (ND) or HFD (60% fat) feeding. After 10 weeks on the HFD, the mice with the *TRPM2* deficiency exhibited a 34.5% increase in body weight and a 50% rise in liver weight compared with their respective *TRPM2*^+/+^ littermates (Figure 1A). The differences in body weight and liver tissue weight between the two strains of mice became even larger when HFD feeding reached 20 weeks (Figure 1A,B).

To investigate the associated liver damage, we measured the plasma aspartate transaminase (AST) and alanine transaminase (ALT) levels between two strains of mice. In the control *TRPM2*^+/+^ mice, the HFD caused a small increase in the plasma AST but not in the plasma ALT. However, in *TRPM2*^−/−^ mice fed with an HFD for 10 weeks, the plasma AST and ALT increased by 67% and 66%, respectively (Figure 1C). After 20 weeks’ HFD feeding, the AST and ALT levels in the *TRPM2*^−/−^ mice further increased to 174% and 143%, respectively (Figure 1C). These results indicate that *TRPM2* knockout exaggerated the HFD-induced liver damage. Thin liver tissue sections were prepared for histological examinations. We stained the thin liver sections with hematoxyline and eosin (H&E) and Oil Red O. Under the HFD, the liver tissue of the *TRPM2*^−/−^ mice showed more extensive signs of lipid accumulation when compared with that of the *TRPM2*^+/+^ mice (Figure 1D,E). These changes also coincided with a large increase in the formation of collagen and fibrotic scar tissue in the liver sections of the *TRPM2*-deficiency mice, as stained with Masson’s Trichrome and Sirius Red (Figure 1D,E). However, under the ND, there was no difference in the histological appearance of the liver tissue between the *TRPM2*^−/−^ mice and the control *TRPM2*^+/+^ mice. These data indicate that *TRPM2* deficiency aggravates hepatic steatosis and fibrosis.

Since the liver is a key organ involved in the regulation of cholesterol metabolism, we next measured the liver and plasma levels of cholesterol. The results showed that the HFD caused an elevation of plasma cholesterol levels, which was more marked in the *TRPM2*^−/−^ mice than in the *TRPM2*^+/+^ mice after 20 weeks of HFD feeding (Figure 1F). The HFD also resulted in higher liver cholesterol levels in the *TRPM2*^−/−^ mice than in the *TRPM2*^+/+^ mice (Figure 1G). These data suggest that *TRPM2* deficiency may induce hypercholesterolemia and higher liver cholesterol accumulation.

Because the HFD for 10 weeks was enough to induce marked hepatic steatosis and fibrosis, in other later experiments, we mostly used a 10-week HFD.

### 3.2. TRPM2 Gene Knockout Disrupted Lipophagy and Reduced Lysosomal Abundance

**Figure 2:**
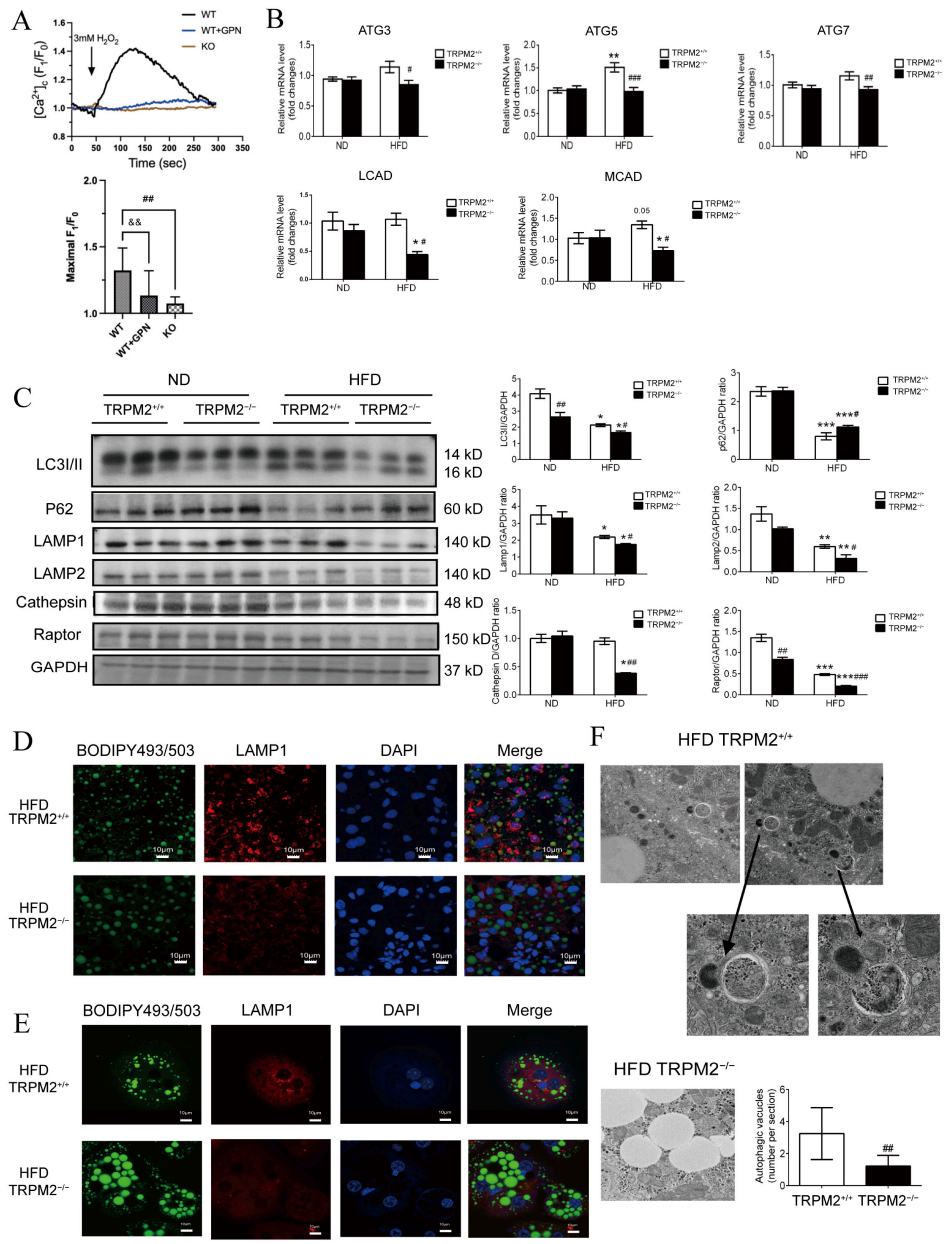
*TRPM2* gene knockout impaired lipophagy and reduced lysosomal abundance. (**A**) H_2_O_2_ induced a cytosolic Ca^2+^ rise mainly by stimulating lysosomal TRPM2 in the primary cultured mouse hepatocytes. Shown are the representative traces (upper panel) and data summary (lower panel) of the cytosolic Ca^2+^ changes in response to 3 mM H_2_O_2_ in the primary cultured hepatocytes bathed in physiological Tyrode’s saline. The H_2_O_2_-induced Ca^2+^ rise diminished with lysosome disruption (200 μM GPN) and with *TRPM2* gene knockout. *N* = 9–26 experiments, 30–40 cells in each experiment. (**B**) mRNA levels of several key lipophagy-regulating genes in the livers of *TRPM2*^+/+^ and *TRPM2*^−/−^ mice fed with an ND or HFD for 10 weeks. *N* = 7–10 mice. (**C**) Representative Western blot images and data summary comparing the expression levels of autophagy markers LC3 and P62, lysosome markers LAMP1 and LAMP2, and Cathepsin D in the livers of *TRPM2*^+/+^ and *TRPM2*^−/−^ mice fed with an ND or HFD for 10 weeks. *N* = 3 mice. (**D**,**E**) Co-localization of BODIPY493/503 (green) with LAMP1 in the liver tissue (**D**) and the primary cultured hepatocytes (**E**) from *TRPM2*^+/+^ and *TRPM2*^−/−^ mice fed with the HFD for 10 weeks. Representative of *N* = 5 mice, 10 slides per mouse. (**F**) Representative TEM images of autophagosomes and data quantification of autophagosomes in the primary cultured hepatocytes from *TRPM2*^+/+^ and *TRPM2*^−/−^ mice fed with an HFD for 10 weeks. The autophagosomes were identified based on the hallmark feature of double-membraned vesicles enclosing morphologically intact cytoplasm, organelles or protein aggregates. Representative of *N* = 3 mice, 10 sections per mouse, 5 images in each section. The two upper images are from *TRPM2*^+/+^ hepatocytes; the middle images are the magnified images from *TRPM2*^+/+^ hepatocytes; and the bottom left image is from *TRPM2*^−/−^ hepatocytes. Data are shown as means ± SEM. Scale bar: 10 µm as indicated. * *p* < 0.05, ** *p* < 0.01, *** *p* < 0.001 ND vs. HFD; ^#^ *p* < 0.05, ^##^ *p* < 0.01, ^###^ *p* < 0.001 *TRPM2*^+/+^ vs. *TRPM2*^−/−^, ^&&^ *p* < 0.01 *TRPM2*^+/+^ vs. *TRPM2*^+/+^ + GPN.

We first determined the functional presence of TRPM2 in hepatocytes by measuring TRPM2-mediated cytosolic Ca^2+^ changes. The primary cultured hepatocyte cells from *TRPM2*^+/+^ and *TRPM2*^−/−^ mice were bathed in physiological Tyrode’s saline. Challenging of the cells with 3 mM H_2_O_2_ induced a cytosolic Ca^2+^ rise in the hepatocytes of the *TRPM2*^+/+^ mice but not those of the *TRPM2*^−/−^ mice (Figure 2A), confirming that the cytosolic Ca^2+^ rise in response to the H_2_O_2_ was mediated by TRPM2. Importantly, after treating the cells with 200 μM glycyl phenylalanine 2-naphthylamide (GPN) to disrupt lysosomes [15], the 3 mM H_2_O_2_-induced cytosolic Ca^2+^ rise in the *TRPM2*^+/+^ hepatocytes was markedly reduced (Figure 2A), suggesting that this Ca^2+^ rise mostly resulted from lysosomal Ca^2+^ release.

To investigate the role of TRPM2 in lipophagy, we firstly compared the liver expressions of several key lipophagy-related genes between the *TRPM2*^+/+^ and *TRPM2*^−/−^ mice with RT-qPCR. Under the HFD conditions, the expression levels of several lipophagy-related proteins, ATG3, ATG5 and ATG7, were found to be lower in the liver samples from the *TRPM2*^−/−^ mice than those from the *TRPM2*^+/+^ mice (Figure 2B). The expression levels of two other genes that are involved in lipid catabolism, long-chain acyl-CoA dehydrogenase *(LCAD)* and medium-chain acyl-CoA dehydrogenase *(MCAD)*, were decreased in the HFD *TRPM2*^−/−^ mice (Figure 2B); Western blot analysis further confirmed the lower expression levels of autophagic markers LC3-II and mTORC1, lysosomal markers LAMP1 and LAMP2, and lipophagy-related lysosomal protease-Cathepsin D, as well as a higher expression of autophagic marker P62, in the liver samples from the *TRPM2*^−/−^ mice than those from the *TRPM2*^+/+^ mice under HFD conditions (Figure 2C). These data suggest that *TRPM2* deficiency inhibits HFD-induced hepatic lipophagy at both the transcriptional and translational levels. Note that the high-fat diet has been reported to decrease GAPDH expression in rats [24]. However, we did not observe any obvious change in GAPDH levels in response to the HFD in our mouse model (Figure 2C).

We next employed fluorescent anti-LAMP1 antibodies and neutral lipid dye BODIPY493/503 to monitor lysosomal abundance and lipid droplets, respectively, in liver tissue sections (Figure 2D) and in the primary cultured hepatocytes (Figure 2E). The results showed that under HFD conditions, the expression of lysosome marker LAMP1 was lower, with concurrent enlargement of the lipid droplet sizes in the liver/hepatocyte samples from the *TRPM2*^−/−^ mice compared with those from the *TRPM2*^+/+^ mice (Figure 2D,E). Transmission electron microscopy (TEM) was used to examine autophagosomes. TEM is the gold standard for visualizing autophagosomes. The results showed that under HFD conditions, *TRPM2*^+/+^ hepatocytes have more autophagosomes than those in *TRPM2*^−/−^ hepatocytes (Figure 2F).

**Figure 3:**
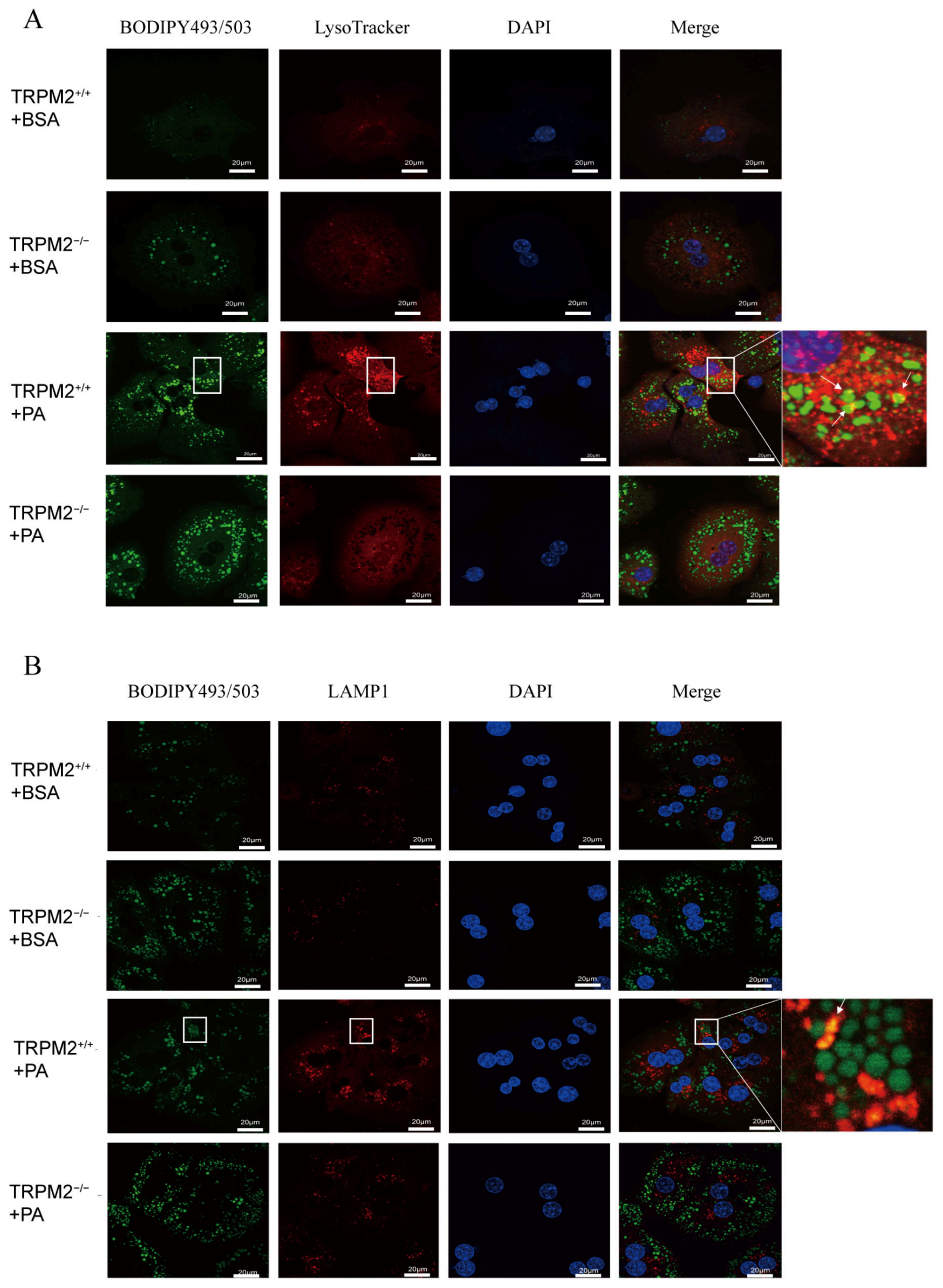
*TRPM2* gene knockout reduced lysosomal abundance in PA-treated primary hepatocytes. The primary cultured hepatocytes from *TRPM2*^+/+^ and *TRPM2*^−/−^ were treated with PA (250 µM for 24 h) to induce lipid accumulation or with BSA as a control. (**A**) The cells were co-stained with lysosome marker LysoTracker and neutral lipid dye BODIPY493/503 or (**B**) with lysosome marker LAMP1 and BODIPY493/503. Scale bar: 20 µm as indicated. Representative of *N* = 10 slides from the cells prepared from 5 mice.

We next established a cultured cell model of NAFLD by incubation with saturated fatty acid palmitic acid (PA) or bovine serum albumin (BSA) as a control [22]. Herein, the primary cultured hepatocytes were prepared from *TRPM2*^+/+^ and *TRPM2*^−/−^ mice under an ND, followed by PA treatment to induce lipid accumulation (Figure 3A,B). The lipid droplets were stained with green fluorescent probe BODIPY493/503, while the lysosomes/autolysosomes were stained with LysoTracker or LAMP1. The PA treatment increased the lipid accumulation in the hepatocytes, as indicated by the BODIPY493/503 staining, with a concurrent increase in lysosomal abundance, as indicated by the LysoTracker or LAMP1 staining (Figure 3A,B). Importantly, the *TRPM2* gene knockout markedly reduced the abundance of lysosomes/autolysosomes in the hepatocytes (Figure 3A,B). Furthermore, some lysosomes/autolysosomes were localized very closely to lipid droplets or even co-localized with lipid droplets in *TRPM2*^+/+^ hepatocytes (Figure 3A,B), which agreed with the functional role of lysosomes/autolysosomes in lipid degradation.

Taken together, the *TRPM2* gene knockout disrupted lipophagy and reduced the abundance of lysosomes and autolysosomes in the hepatocytes.

### 3.3. TRPM2 Gene Knockout Inhibited TFEB Expression and Its Nuclear Translocation

**Figure 4:**
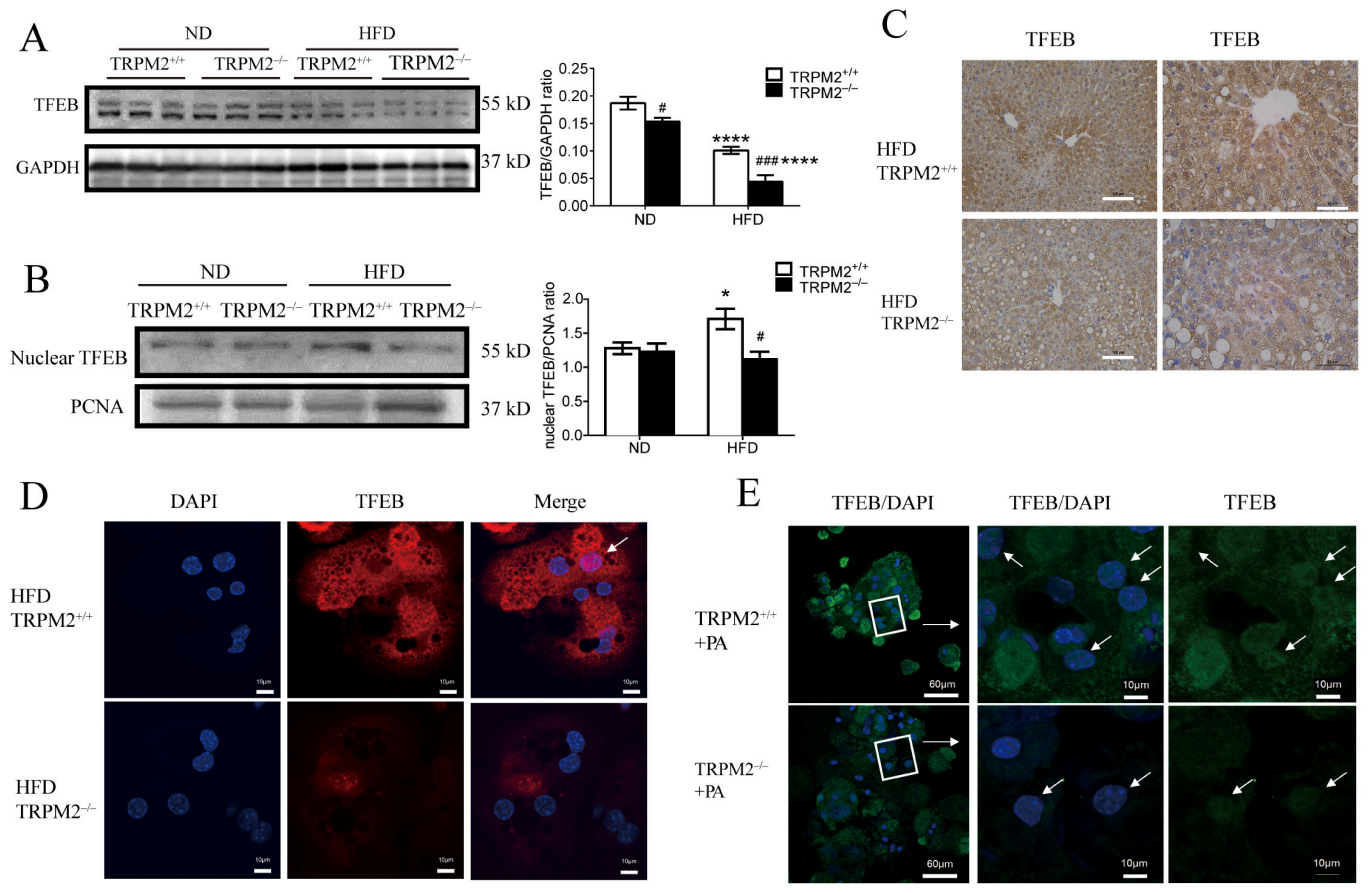
*TRPM2* gene knockout inhibited TFEB expression and its nuclear translocation. (**A**,**B**) Representative Western blot images and data summary comparing the expression levels of total TFEB (**A**) and nuclear TFEB (**B**) in the livers of *TRPM2*^+/+^ and *TRPM2*^−/−^ mice fed with ND or HFD for 10 weeks. *N* = 3 mice. (**C**) Immunohistochemical staining of TFEB in the liver tissue sections of *TRPM2*^+/+^ and *TRPM2*^−/−^ mice fed with an HFD for 10 weeks. Representative of *N* = 5 mice, 10 slides per mouse. (**D**) Immunofluorescent staining of TFEB (in red fluorescence) in the primary cultured hepatocytes from *TRPM2*^+/+^ and *TRPM2*^−/−^ mice fed with an HFD for 10 weeks. Representative of *N* = 10 slides from the cells prepared from 5 mice. (**E**) Immunofluorescent staining of TFEB (in red fluorescence) in the primary cultured hepatocytes from *TRPM2*^+/+^ and *TRPM2*^−/−^ mice fed with ND. The cells were incubated with 250 µM PA for 24 h before the immunostaining. *N* = 10 slides from the cells prepared from 5 mice. The white arrows in (**D**,**E**) show the nuclear localization of the TFEB. * *p* < 0.05, **** *p* < 0.0001 ND vs. HFD; # *p* < 0.05, ### *p* < 0.001 *TRPM2*^+/+^ vs. *TRPM2*^−/−^.

We examined the possible involvement of TFEB, which is a master regulator of lysosomal biogenesis and autophagy/lipophagy [25,26]. Western blot analysis showed that under the HFD, TFEB expression in liver tissues was lower in *TRPM2*^−/−^ mice than in *TRPM2*^+/+^ mice (Figure 4A). Next, nuclear proteins were extracted from liver tissues and analyzed for TFEB expression. The results showed that with the ND, there was no difference in nuclear TFEB expression between the *TRPM2*^−/−^ and *TRPM2*^+/+^ mice. However, under the HFD, the expression of nuclear TFEB was much higher in the *TRPM2*^+/+^ mice than in the *TRPM2*^−/−^ mice (Figure 4B).

Immunostaining of thin liver tissue sections with anti-TFEB antibodies further confirmed the higher expression of TFEB in the *TRPM2*^+/+^ livers than in the *TRPM2*^−/−^ livers (Figure 4C). TFEB immunostaining was also performed in the primary cultured hepatocytes from the *TRPM2*^+/+^ mice and *TRPM2*^−/−^ mice after the HFD (Figure 4D). In another series of experiments, the primary cultured hepatocytes were prepared from the *TRPM2*^+/+^ mice and *TRPM2*^−/−^ mice under an ND, followed by PA treatment to induce lipid accumulation (Figure 4E). Under both the HFD and PA treatments, TFEB expression and nuclear translocation were both higher in the primary cultured hepatocytes from the *TRPM2*^+/+^ mice than those from the *TRPM2*^−/−^ mice (Figure 4D,E). Together, these data demonstrate that the *TRPM2* gene knockout reduced the expression and nuclear translocation of TFEB, suggesting that TRPM2 may act through TFEB to regulate lysosomal biogenesis and autophagy.

### 3.4. TRPM2 Gene Knockout Reduced Lysosomal LAL Expression/Activity

**Figure 5:**
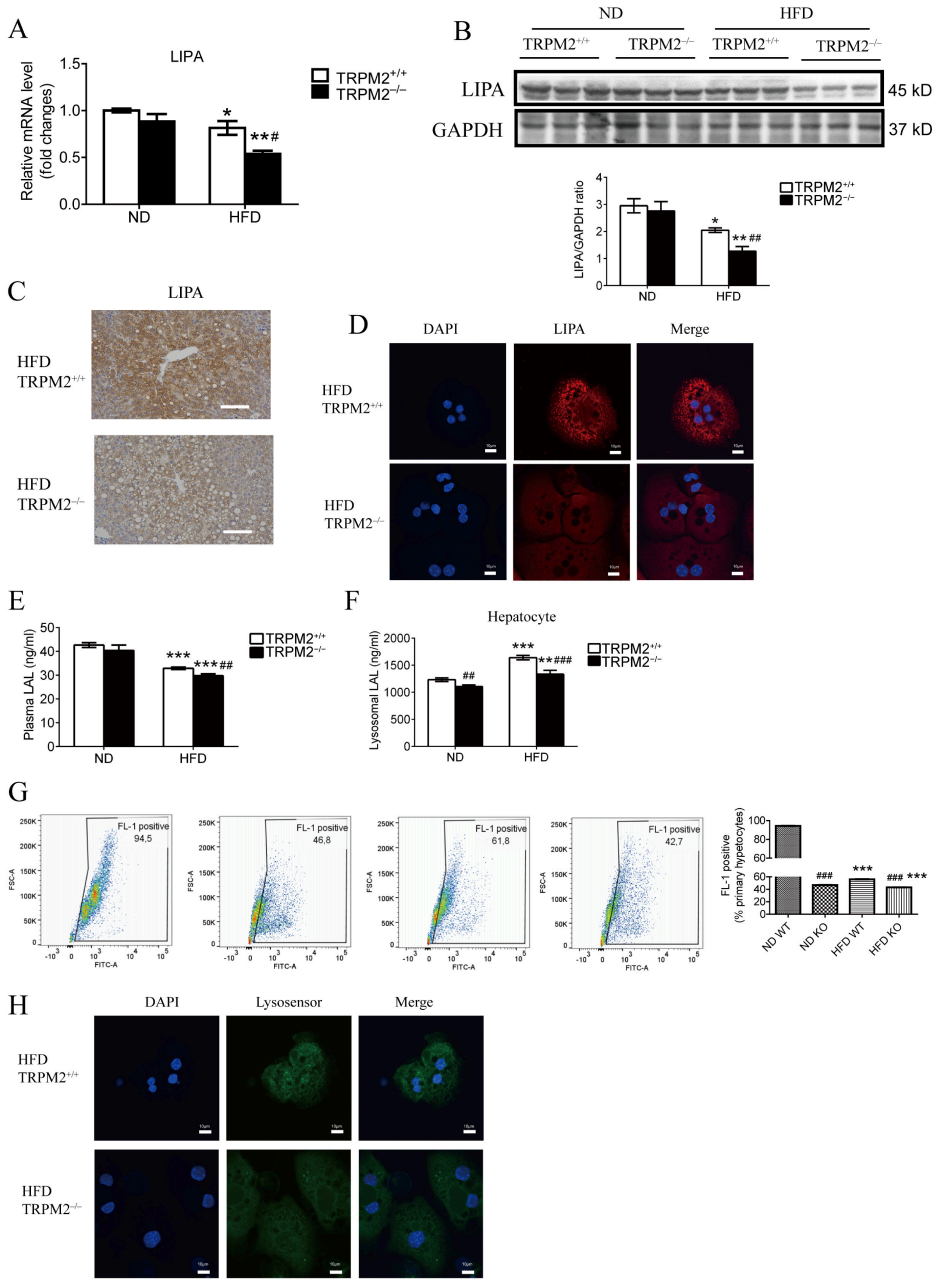
*TRPM2* gene knockout reduced lysosomal LAL expression/activity. (**A**) mRNA levels of *LIPA* in the livers of *TRPM2*^+/+^ and *TRPM2*^−/−^ mice fed with an ND or HFD for 10 weeks. *N* = 7–10 mice. (**B**) Representative Western blot images and data summary comparing the expression of LIPA proteins in the livers of *TRPM2*^+/+^ and *TRPM2*^−/−^ mice fed with an ND or HFD for 10 weeks. *N* = 3 mice. (**C**) Immunohistochemical staining of LIPA proteins in the liver tissue sections of *TRPM2*^+/+^ and *TRPM2*^−/−^ mice fed with an HFD for 10 weeks. Representative of *N* = 5 mice, 10 slides per mouse. (**D**) Immunofluorescent staining of LIPA in the primary cultured hepatocytes from *TRPM2*^+/+^ and *TRPM2*^−/−^ mice fed with an HFD for 10 weeks. Representative of *N* = 10 slides from the cells prepared from 5 mice. (**E**,**F**) Plasma LAL (**E**) and hepatocyte lysosomal LAL (**F**) activity in *TRPM2*^+/+^ and *TRPM2*^−/−^ mice fed with an ND or HFD for 10 weeks. *N* = 6–8 mice. (**G**) Flow cytometer-based measurement of LAL enzyme activity in the primary hepatocytes from *TRPM2*^+/+^ and *TRPM2*^−/−^ mice under ND or HFD conditions. *N* = 5 repeats from the cells prepared from 5 mice. (**H**) Representative fluorescent images of LysoSensor-based lysosomal/autolysosomal acidification in the primary hepatocytes from *TRPM2*^+/+^ and *TRPM2*^−/−^ mice fed with an HFD for 16 weeks. *N* = 10 repeats from the cells prepared from 5 mice. Data are shown as means ± SEM. Scale bar: 100 µm or 60 µm or 10 µm as indicated. * *p* < 0.05, ** *p* < 0.01, *** *p* < 0.001 ND vs. HFD; # *p* < 0.05, ## *p* < 0.01, ### *p* < 0.001 *TRPM2*^+/+^ vs. *TRPM2*^−/−^.

Lysosomal acid lipase (LAL), encoded by *LIPA*, is an essential lysosomal enzyme that plays a key role in lipophagy [27,28]. Therefore, we next examined the role of LAL. RT-qPCR and Western blot analysis showed that under ND, *TRPM2* knockout did not alter the expression of *LIPA*. However, under HFD, *TRPM2* knockout markedly reduced the expression of LIPA at both the mRNA (Figure 5A) and protein levels (Figure 5B). Immunostaining of thin liver tissue sections (Figure 5C) and the primary cultured hepatocytes (Figure 5D) with an anti-LIPA antibody further confirmed the higher expression of LIPA in *TRPM2*^+/+^ hepatocytes than in *TRPM2*^−/−^ hepatocytes under HFD conditions.

Enzymatic activity of LAL was also measured. The results showed that under the HFD, plasma LAL (Figure 5E) and lysosomal LAL (Figure 5F) in hepatocytes were both lower in *TRPM2*^−/−^-knockout mice than in *TRPM2*^+/+^ mice. Finally, we evaluated the activity of LIPA by using flow cytometry. The primary hepatocytes were isolated from HFD-treated *TRPM2*^+/+^ and *TRPM2*^−/−^ mice, followed by fluorescent labeling with a LysoLive^TM^ Lysosomal Acid Lipase Assay Kit to monitor LAL activity. The flow cytometry results indicated that *TRPM2* gene deletion reduced the LAL activity in hepatocytes under both the ND and HFD, as indicated by reduced FL-1 positive cells (Figure 5G). Overall, *TRPM2* deletion reduced the expression of LIPA at mRNA and protein levels and also reduced the enzymatic activity of LAL.

Because lysosomal acidification is crucial for lysosomal enzyme activity, we also used LysoSensor Green DND-189 to evaluate HFD-induced lysosomal/autolysosomal acidification in the primary cultured hepatocytes. The results showed an impaired lysosomal/autolysosomal acidification in *TRPM2*^−/−^ hepatocytes compared to *TRPM2*^+/+^ hepatocytes (Figure 5H).

**Figure 6:**
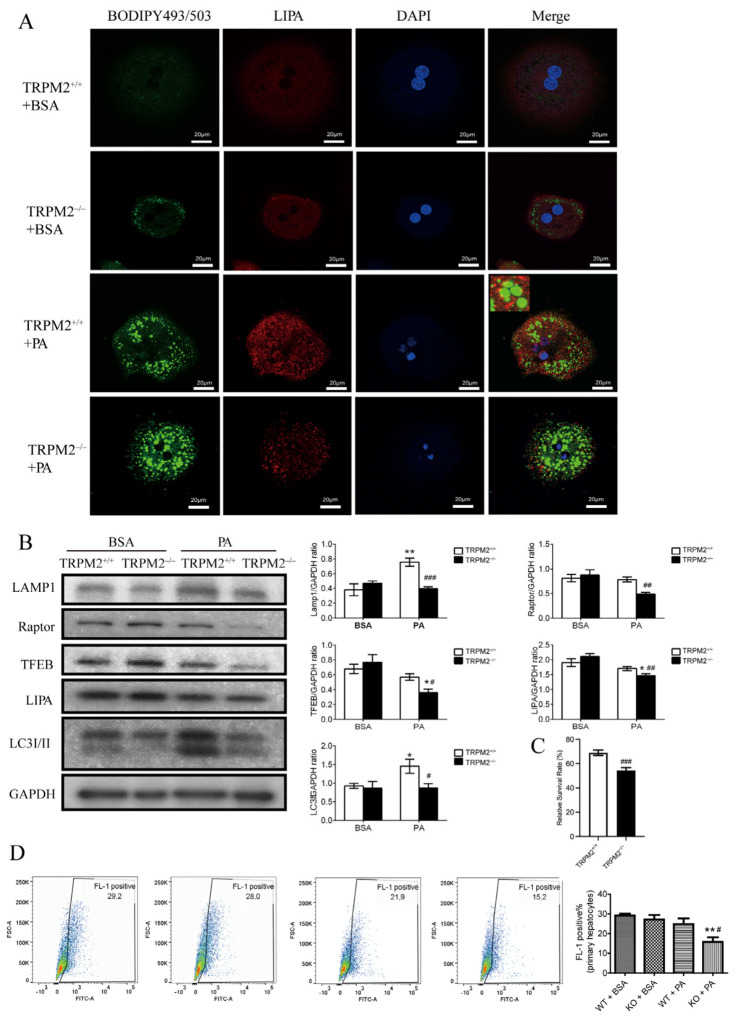
*TRPM2* gene knockout reduced lysosomal LAL expression/activity and lipophagy in PA-treated primary hepatocytes. (**A**) The primary cultured hepatocytes were treated with PA (250 µM for 24 h) to induce lipid accumulation or with BSA as a control, then co-immunofluorescently stained with anti-LIPA antibodies and neutral lipid dye BODIPY493/503. Representative of *N* = 10 slides from the cells prepared from 5 mice. (**B**) Representative Western blot images and data summary comparing the expression levels of LC3, LAMP1, LIPA and TFEB in the primary hepatocytes incubated with PA (250 µM for 48 h) or BSA as a control. *N* = 3 mice. (**C**) The relative survival rate was calculated as the ratio of live cell counts in the PA-treated group to those in the BSA-treated control group. *N* = 4 repeats. (**D**) Flow cytometer-based measurement of LAL enzyme activity in the primary hepatocytes incubated with (250 µM PA for 48 h) or BSA as a control. *N* = 5 repeats from the cells prepared from 5 mice. Data are shown as means ± SEM. Scale bar: 20 µm as indicated. * *p* < 0.05, ** *p* < 0.01 BSA vs. PA; # *p* < 0.0,5 ## *p* < 0.01, ### *p* < 0.001 *TRPM2*^+/+^ vs. *TRPM2*^−/−^.

We next utilized the cultured cell model of MASLD/NAFLD by PA treatment [22]. The primary cultured hepatocytes were prepared from *TRPM2*^+/+^ mice and *TRPM2*^−/−^ mice under an ND, followed by PA treatment to induce lipid accumulation. The PA treatment increased the expression of LIPA in the hepatocytes, as indicated by LIPA fluorescent immunostaining, with a concurrent increase in lipid accumulation as indicated by BODIPY493/503 staining (Figure 6A). *TRPM2* gene knockout reduced the LIPA expression in the PA-treated hepatocytes (Figure 6A). Western blot analysis was used to further examine the role of TRPM2 in lipophagic indexes in the PA-treated hepatocytes. The results showed that *TRPM2* knockout reduced the expressions of LAMP1, Raptor, TFEB, LC3-II and LIPA in the PA-treated hepatocytes (Figure 6B). Trypan blue staining was performed to assess cell viability following 48 h of PA treatment (Figure 6C). A flow cytometry-based assay of lysosomal acid lipase activity further confirmed that *TRPM2* knockout reduced the LAL activity in the PA-treated hepatocytes (Figure 6D). These results agree well with those from the HFD-treated hepatocytes.

## 4. Discussion

Ca^2+^ signaling plays an important role in lipid metabolism in hepatocytes. An abnormality of Ca^2+^ signaling may aggravate lipid accumulation in steatotic hepatocytes. One well-documented example is the store-operated Ca^2+^ entry mediated by Orai1 channels [29]. Lipids inhibit store-operated Ca^2+^ entry to alter intracellular Ca^2+^ homeostasis, which in turn increases lipid synthesis and decreases lipolysis, resulting in aggravated lipid accumulation in steatotic hepatocytes [29]. The endolysosomal Ca^2+^-permeable channel TRPML1 may also regulate lipid accumulation. Mutation of TRPML1 causes a rare genetic disease, Mucolipidosis type IV, which is characterized by accumulation of lipids and proteins in cytoplasmic vacuoles derived from lysosomes [10]. In the present study, we found that functional TRPM2, which is activated by H_2_O_2_, predominantly mediates lysosomal Ca^2+^ release in hepatocytes, and the channels play an important role in lipid metabolism in steatotic hepatocytes. A mouse model of MASLD/NAFLD was established using an HFD [23]. Knockout of the *TRPM2* gene was found to aggravate the lipid accumulation and fibrosis in the liver tissues of these mice. The *TRPM2* gene knockout also aggravated the HFD-induced liver damage as indicated by elevated plasma AST and ALT levels. These data demonstrate that *TRPM2* deficiency may aggravate liver steatosis and MASLD/NAFLD under HFD conditions.

*TRPM2* is known to regulate autophagy. The activity of *TRPM2* may either promote autophagy or inhibit autophagy, dependent on different cell types [15,16,17,18,19]. However, it remains unclear whether *TRPM2* may regulate lipophagy. In the present study, RT-qPCR and Western blot analyses showed that *TRPM2* knockout reduced lipophagy, as indicated by a reduced LC3-II level, an increased P62 level, and a decreased expression of lipophagy-related proteins (ATG3, ATG5 and ATG7). Cellular immunostaining experiments showed reduced expression of LAMP1 and LAMP2 in *TRPM2* knockout hepatocytes, which was confirmed by Western blots, suggesting that the *TRPM2* knockout decreased the lysosome biogenesis under HFD conditions. Transmission electron microscopy also confirmed a reduced number of autophagosomes in the hepatocytes of the *TRPM2*-knockout mice. Together, these data provide strong evidence that *TRPM2* knockout decreases lipophagy, causing lipid accumulation in hepatocytes.

We further explored the underlying mechanisms of how *TRPM2* knockout could reduce lysosome biogenesis and lipophagy. TFEB is a master regulator of lysosomal biogenesis and autophagy/lipophagy [25,26]. The subcellular localization and activity of TFEB are regulated by mechanistic target of rapamycin (mTOR)-mediated phosphorylation. Dephosphorylated TFEB translocates to the nucleus to induce the transcription of key genes that can control lysosomal biogenesis and lipophagy [25,26]. Interestingly, previous studies have shown that endolysosomal Ca^2+^-permeable channel TRPML1 can activate calcineurin to dephosphorylate TFEB, causing nuclear translocation of TFEB and consequently modulating lysosomal biogenesis and autophagy [7,10,30,31]. Furthermore, once in the nucleus, *TFEB* binds to its own promoter**,** directly driving the transcription of the *TFEB* gene, forming a Ca^2+^-driven positive feedback loop to ensure a steady, increased supply of newly synthesized TFEB protein for prolonged lysosomal biogenesis and autophagic flux [26,32]. In our study, Western blot and cellular immunostaining experiments showed that *TRPM2* gene knockout reduced the expression and nuclear translation of TFEB. These results suggest that TRPM2 may act through TFEB to regulate lysosomal biogenesis and lipophagy, likely via a mechanism similar to that of TRPML1.

Lysosomal acid lipase (LAL), encoded by the *LIPA* gene, is an essential lysosomal enzyme in the lipophagic process. LAL hydrolyses cholesterol ester and triglycerides delivered to the lysosome, generating free fatty acids and free cholesterol [27,33]. Loss of function mutation or decreased activity of LAL may cause accumulation of cholesterol ester and triglycerides in the liver, favoring hepatic steatosis [27,28,33]. In our study, Western blots and cellular immunostaining both showed that under HFD conditions, the expression level of LIPA was lower in the *TRPM2* knockout hepatocytes than in wild-type hepatocytes. Measurement of LAL enzymatic activity also indicated that under HFD conditions, plasma LAL and hepatocyte lysosomal LAL were both lower in *TRPM2*-knockout mice than in wild-type mice. Similarly, in the primary cultured hepatocytes treated with saturated fatty acid PA, the expression and activity of LAL were also lower in *TRPM2* knockout hepatocytes than in wild-type hepatocytes. These data provide direct evidence that *TRPM2* knockout reduces the expression/activity of LAL to suppress lipophagy, consequently causing lipid accumulation and hepatic steatosis. Consistently, we also showed that *TRPM2*-knockout mice had decreased lysosomal abundance and less lysosomal acidification, both of which are expected to reduce LAL activity in hepatocytes.

The present study has demonstrated that *TRPM2* deficiency impairs lipophagy to aggravate the HFD-induced hepatic steatosis and liver injury. In other words, TRPM2 itself has a protective role against HFD-induced hepatic steatosis. Similar protective effects of TRPM2 have been reported in other diseases. For example, TRPM2 promotes autophagy to protect against cisplatin-induced acute kidney injury [34]. TRPM2 also has a protective role in ischemia reperfusion-mediated cardiovascular damage via its action on mitochondria [35,36]. In addition, TRPM2 protects against the radiation-induced DNA damage response [37], Escherichia coli sepsis and pneumonic bacterial infection [38,39]. However, also note that TRPM2 may play a detrimental role in some other diseases due to its linkage to ROS production and apoptotic cell death, such as in Alzheimer’s disease, ischemic brain injury and ROS-mediated cardiomyocyte death [12]. TRPM2 activity may also promote vascular inflammation and atherosclerosis [14]. In the liver, TRPM2 has previously been reported to mediate ROS- and acetaminophen-induced liver injury [17,40,41]. Therefore, whether TRPM2 has a beneficial role or detrimental role is dependent on the disease type and on which mechanism has the predominate effect. In the liver, TRPM2 may mediate ROS- and acetaminophen-induced liver injury, but it protects against HFD-induced hepatic steatosis and injury. In our model, the detrimental effect of lipophagy impairment in *TRPM2* KO mice overrode the potential beneficial effect of anti-inflammation, causing overall aggravation of hepatic steatosis and liver injury.

In summary, our present study demonstrated a critical role of TRPM2 channels in the regulation of lysosome-mediated lipophagy in HFD mice. *TRPM2* deficiency may reduce the expression and nuclear translocation of TFEB, decrease lysosomal biogenesis and inhibit LAL expression/activity, consequently impairing hepatic lipophagy, resulting in hepatic steatosis. This study highlights the possibility of targeting TRPM2 channels as a therapeutic target in MASLD/NAFLD.

## Data Availability

The data presented in this study are available on request from the corresponding author due to privacy.
